# Validation of EBUS-TBNA-integrated nodal staging in potentially node-positive non-small cell lung cancer

**DOI:** 10.1007/s11748-013-0263-z

**Published:** 2013-06-09

**Authors:** Yuichi Sakairi, Hidehisa Hoshino, Taiki Fujiwara, Takahiro Nakajima, Kazuhiro Yasufuku, Shigetoshi Yoshida, Ichiro Yoshino

**Affiliations:** 1 Department of General Thoracic Surgery, Chiba University Graduate School of Medicine, 1-8-1, Inohana, Chiba, 260-8670 Japan; 2 Division of Thoracic Surgery, Toronto General Hospital, University Health Network, University of Toronto, Toronto, Canada

**Keywords:** EBUS-TBNA, Staging, Lung cancer, FDG-PET, Lymph nodes

## Abstract

**Objective:**

Nodal staging of lung cancer is important for selecting surgical candidates. Endobronchial ultrasound-guided transbronchial needle aspiration (EBUS-TBNA) was evaluated as a modality for nodal staging of patients with potentially node-positive non-small cell lung cancer (NSCLC).

**Methods:**

Endobronchial ultrasound-guided transbronchial needle aspiration was used for nodal staging of NSCLC patients with radiological N2/3 disease (short axis >10 mm on computed tomography and/or standardized positron emission uptake value >2.5 on 2-deoxy-2[F-18] fluoro-d-glucose positron emission tomography), T-stage ≥ T2, or positive serum carcinoembryonic antigen. Data on eligible patients were extracted from the database of our institution and analyzed for differences in nodal stages between radiological staging (RS) and EBUS-TBNA-integrated staging (ES), with validation by pathological staging of patients who had undergone surgery.

**Results:**

Of 480 eligible patients, there were 135 N0/1 and 345 N2/3 patients according to RS. Out of the 345 patients staged as N2/3 by RS, 113 (33 %) were downgraded to N0/1 by ES. Out of the 135 patients staged as N0/1 by RS, 12 (9 %) were upgraded to N2/3 by ES. Patients were restaged as N0/1 in 236 cases and N2/3 in 244 cases by ES, and the distributions of nodal stage between RS and ES were significantly different (*p* < 0.001). Finally, 215 out of the 236 ES-N0/1 patients underwent lung resection, and 195 (90.7 %) and 20 patients were staged by pathology as N0/1 and N2, respectively.

**Conclusions:**

Endobronchial ultrasound-guided transbronchial needle aspiration is more accurate for lymph node staging compared to radiological staging. EBUS-TBNA can identify patients who are true candidates for surgery.

## Introduction

Mediastinal nodal staging of patients with non-small cell lung cancer (NSCLC) affects patient outcome, since initial surgery is not indicated for patients with clinical manifestations of N2 or N3 (N2/3) disease [1, 2]. Mediastinoscopy has been the gold standard for mediastinal histological nodal staging, providing satisfactory sensitivity (74–92 %), and specificity (100 %) despite its invasiveness [3]. Alternatively, 2-deoxy-2[F-18]fluoro-d-glucose positron emission tomography (FDG-PET) and PET-computed tomography (CT) have been used as non-invasive mediastinal nodal staging methods with a reported sensitivity of 79–85 % and specificity of 90–91 % [4]. However, FDG-PET can over-stage patients with reactive lymph nodes showing elevated FDG accumulation, and this staging error can lead to reduced numbers of surgical candidates.

Endobronchial ultrasound-guided transbronchial needle aspiration (EBUS-TBNA) is an established modality used for preoperative lymph node staging of lung cancer [5]. It is a safe, minimally invasive, and validated procedure [6, 7]. EBUS-TBNA has been reported to have a high sensitivity (93 %) and specificity (100 %), and a cohort study comparing EBUS-TBNA, CT, and FDG-PET for lymph node staging of lung cancer found positive predictive values of 100, 37, and 46.5 %, respectively [8]. The efficacy of nodal staging using EBUS-TBNA was validated by meta-analysis, which reported sensitivities ranging from 88 to 93 % and a specificity of 100 % [9, 10].

We studied the efficacy of EBUS-TBNA with respect to the utility of preoperative nodal staging in a case series in a single institution by comparing imaging modalities.

## Methods

### Patients

From January 2004 to December 2009 (6 years), 1327 patients underwent EBUS-TBNA for various indications at Chiba University Hospital. Data on eligible patients were extracted from the database at our institution. The case selection criteria were as follows: (1) patients with NSCLC or suspected NSCLC; (2) no previous treatments for thoracic malignancies; and (3) EBUS-TBNA performed according to the conventional indication to evaluate mediastinal lymph nodes in patients staged either N2/N3 using radiological modalities (CT and/or FDG-PET), or according to an extended indication in patients staged N0/N1 using radiological modalities, with T-stage ≥ T2 or positive serum carcinoembryonic antigen (CEA) (>5 ng/dL in our hospital laboratory). These criteria are listed in Table 1. Positive radiological criteria consisted of lymph nodes with a short axis >10 mm on enhanced CT, or a nodal standardized uptake value >2.5 on FDG-PET. Lymph node stations and numbers were determined according to the seventh edition of the TNM classification for lung cancer (UICC-7); old data staged according to UICC-6 were re-evaluated and revised to UICC-7 stations and numbers [11].

**Table 1:** Inclusion criteria

Non-small cell lung cancer (NSCLC) or suspected NSCLC
No previous treatments for thoracic malignancies
EBUS-TBNA performed for the evaluation of mediastinal lymph nodes and
N2/N3 diagnosed by radiological modality (CT and/or PET)
N0/N1 diagnosed by radiological modality with advanced T stage (≥T2) or positive serum CEA

EBUS-TBNA indicates endobronchial ultrasound-guided transbronchial needle aspiration

*CT* computed tomography, *PET* positron emission tomography, *CEA* carcinoembryonic antigen

### Endobronchial ultrasound-guided transbronchial needle aspiration

Endobronchial ultrasound-guided transbronchial needle aspiration was performed on lymph nodes with a short axis ≥5 mm on chest CT or that were positive on FDG-PET. The procedures were performed at Chiba University Hospital by well-trained thoracic surgeons or trainees under supervision. EBUS-TBNA was performed in an outpatient setting. Patients received laryngeal anesthesia (1 % lidocaine nebulizer and 4 % lidocaine spray) prior to the examination. Midazolam (1–3 mg/body) was injected intravenously for conscious sedation and additional local anesthesia (1 % lidocaine) was administered from the fiberscope as needed during the examination. The convex-probe EBUS (BF-UC260F-OL8, Olympus, Japan) and a dedicated ultrasound scanner (EU-C2000/EU-C60; Olympus) were used for nodal observation. Histological and cytological specimens were obtained using a dedicated 22-gauge needle equipped with an internal stylet (NA-201SX-4022; Olympus). Cytological specimens were smeared onto glass slides, and after the slides had been dried, they were immediately stained with Diff-Quik (Sysmex Corporation; Kobe, Japan). On-site screening was performed by a cytoscreener who decided if the slide yielded diagnostic information. Final cytological and histological diagnoses were established by pathologists. We did not apply EBUS-TBNA to inaccessible nodal stations five (subaortic nodes), six (paraaortic nodes), eight (paraesophageal nodes), and nine (pulmonary ligament nodes). We performed a systematic nodal inspection for selected patients; lymph nodes were visualized beginning with N1 lymph nodes, followed by N2 nodes, and finally, N3 nodes. EBUS-TBNA was then first performed on N3 nodes, followed by N2 nodes, and, if needed, by N1 nodes. If N3 nodes were found to be positive for malignancy by rapid on-site cytological evaluation, we terminated the procedure [12].

### Clinical and surgical staging

Extracted data were evaluated as follows: the differences in nodal stages determined by radiological staging (RS) versus EBUS-TBNA integrated staging (ES), which was based on EBUS-TBNA and CT and/or FDG-PET, were analyzed and verified against the pathological stages of specimens from patients (N0/N1 diagnosed by the EBUS-TBNA integrated staging system) who had undergone surgery. In addition, only FDG-PET was used for nodal staging on EBUS-TBNA inaccessible nodes in ES. The efficacy of EBUS-TBNA was defined by its diagnostic accuracy with respect to finding N2/3 patients. In this study, we regarded the clinical N0/1 patients as having an indication for surgery.

### Statistical analysis

Frequency analysis was performed using the Chi square test. Data were analyzed using JMP 9 (SAS Institute Inc., Cary, NC). All *p* values were based on a two-tailed hypothesis test; a *p* value of <0.05 was considered to have statistical significance.

### Ethics committee approval

The ethics committee of Chiba University Graduate School of Medicine approved this research (No. 1315).

## Results

### Patients

Among 1327 patients who had undergone EBUS-TBNA, 480 patients were eligible for analysis. Data were extracted for 376 men and 104 women, with a mean age of 67.1 years. Histological diagnoses from this patient cohort consisted of adenocarcinoma in 291 (61 %), squamous cell carcinoma in 115 (24 %), other histological subtype of NSCLC in 30 (6 %), and NSCLC, not otherwise specified in 44 (9 %) patients. The average time interval between EBUS-TBNA and surgical resection was 27.7 ± 15.4 days.

### Nodal staging by RS, ES, and surgical pathology

**Fig. 1:**
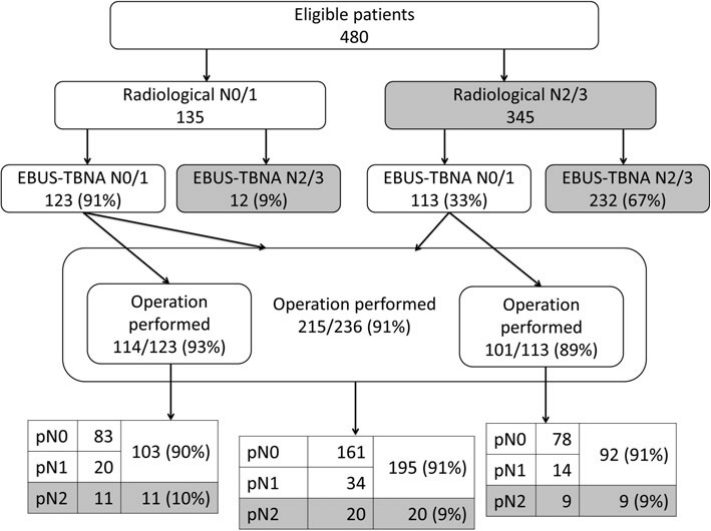
Differences in nodal stages determined by radiological, EBUS-TBNA-integrated, and surgical pathological staging

The numbers of patients classified according to nodal stage determined by RS, ES, and surgical pathologic staging are shown in Fig. 1. Out of 480 patients, RS staged 135 and 345 respective patients with N0/1 and N2/3 disease. Of the 135 patients with N0/1 disease by RS but with a T stage > T2 or positive serum CEA, 12 patients (9 %) were upgraded by EBUS-TBNA from N0/1 to N2/3 disease. Out of the 345 patients with N2/3 disease by RS, 232 patients (67 %) were confirmed to have N2/3 disease and 113 (33 %) were downgraded to N0/1 disease by ES. As a result of ES, 236 patients were restaged as N0/1 disease and 244 as N2/3 disease. Out of the 236 patients with ES-N0/1 disease, 215 underwent surgery. For the remaining 21 patients, the operation was not performed in our institution because of referral to other institutions (13 patients), severe complications (4 patients), or a severe performance status for surgery (4 patients). There were 161, 34, and 20 respective patients with final pathologic nodal stages of pN0, pN1, and pN2. Therefore, 9 % (20/215) of N0/1 surgical patients were diagnosed with pN2 disease based on surgical pathology. In addition, we found three patients with nodal metastases in EBUS-TBNA inaccessible nodes (#5,6,8,9) in RS; all of these patients were diagnosed as cN2 by EBUS-TBNA from other N2 nodes and did not undergo surgery.

Surgical resection was performed on 101 patients (89 %) who were downstaged to N0/1 from N2/3 by ES. There were 78, 14, and 9 respective patients with final pathologic nodal stages of pN0, pN1, and pN2. Therefore, out of 101 patients, 92 patients (91 %) were diagnosed with pN0/1 disease after surgery, and the remaining 9 patients (9 %) had false-negative pN0/1 disease by EBUS-TBNA integrated nodal staging.

Thus, staging by RS diagnosed N0/1 and N2/3 disease in 28.1 and 71.9 % of patients, respectively, whereas ES of these patients diagnosed N0/1 and N2/3 disease in 49.2 and 50.8 %, respectively. These distributions were significantly different (Chi square test: *p* < 0.001). ES led to stage changes in 125 patients (26.0 %) staged by RS, including 12 to higher stages and 113 to lower stages.

In total, we found 20 ES-underdiagnosed patients through this survey. The reasons for the underdiagnoses were micrometastatic disease (7 cases) and technical problems (13 cases) manifested as a negative biopsy result from non-metastatic nodes in the same station (4 cases), or metastasis to a non-biopsied station (6 cases), or EBUS-TBNA inaccessible nodes (3 cases). Additionally, the 5-year survival rate of this pN2 cohort was 40.8 % (Kaplan–Meier method), and the median follow-up period was 62.8 months.

### Statistical analyses

For the 459 cases that consisted of 244 inoperable cases according to ES and 215 surgically resected cases, the sensitivity, specificity, and diagnostic accuracy were calculated despite the limited cohort. If the decision to operate was based only on radiological staging, surgery was performed in 114 radiological N0/1 cases, and 9 cases were eliminated as inoperable; 81.7 % (103/126) of radiological N0/1 cases were confirmed as true N0/1. Then, the estimated sensitivity, specificity, and accuracy of RS were 91.3, 52.8, and 74.9 %, respectively (Table 2). In contrast, the sensitivity, specificity, and accuracy of ES were 92.4, 100, and 95.6 %. We applied an extended indication for EBUS-TBNA (>T2 and high serum CEA level) in N0/1 cases of the RS patients. If we applied conventional EBUS-TBNA criteria (only for radiologically positive nodes) for this cohort, the sensitivity, specificity, and accuracy were 87.9, 100, and 92.4 %, respectively. Thus, the extended criteria for EBUS-TBNA (criteria on Table 1) raised the sensitivity by 4.5 % and the accuracy by 2.6 %, compared with conventional criteria. The differences between the numbers of patients staged by each criterion are shown in Table 2.

**Table 2:** Differences in results of radiological staging versus EBUS-TBNA integrated staging

	Pathological staging		Total
	N0/1	N2/3	
Radiological staging (RS)			
N0/1	103	23	126
N2/3	92	241	333
Conventional^a^ EBUS-TBNA integrated staging			
N0/1	195	32	227
N2/3	0	232	232
Extended^b^ EBUS-TBNA integrated staging (ES)			
N0/1	195	20	215
N2/3	0	244	244
Total	195	264	459

^a^Conventional: EBUS-TBNA applied only in radiologically node-positive cases

^b^Extended: EBUS-TBNA criteria of Table 1 (including radiologically node-negative cases)

## Discussion

In this study, the identification of appropriate surgical lung cancer patients using the EBUS-TBNA integrated staging system led to an increase in the total number of surgical candidates. Our findings show that as high as 33 % of the radiologically N2/3 patients proved to have pN0/1 disease, and they suggest that these patients may undergo inappropriate, inadequate treatment unless EBUS-TBNA is performed.

Thus, a discrepancy between RS and ES existed. Among the 135 RS-N0/1 patients, 12 were ES-diagnosed as N2/3. In the remaining 123, 114 underwent surgery. Of those, 11 were diagnosed as having N2 disease, and 103 (76 %) were surgically proven as N0/1. Nodal status was not specifiable in the 9 ES-N0/1 patients not undergoing surgery. There were at least 23 (17 %) pathological N2/3 patients and 32 (24 %) at the maximum. Therefore, the radiological underdiagnosis rate was somewhere between 17 and 24 %. Likewise, of the 345 RS-N2/3 patients, 232 ES-N2/3 and 9 surgically diagnosed N2 patients were truly N2/3 (241 in total, 70 %). There were 92 surgically proven N0/1 patients, which was 27 % of the RS-N2/3 patients. Therefore, the true overdiagnosis rate was somewhere between 27 and 30 %. This radiological over- or underdiagnosis would lead to inappropriate treatment.

Surgical staging, including mediastinoscopy, has been the standard method for discriminating N2 patients from surgical candidates. However, there has been a paradigm shift in invasive staging. The ASTER study demonstrated that a combination of surgical staging and endosonography (EBUS-TBNA and transesophageal ultrasound-guided fine-needle aspiration) could reduce the number of unnecessary thoracotomies compared with surgical staging alone [13]. More recently, a prospective study of potentially resectable lung cancer patients undergoing EBUS-TBNA followed by mediastinoscopy showed that there were no significant differences in the diagnostic yield between the two procedures when performed by trained thoracic surgeons in a controlled environment. This study indicates that EBUS-TBNA can potentially replace mediastinoscopy in patients with potentially resectable NSCLC [14]. In our series, mediastinoscopy was not performed, and the EBUS-TBNA integrated staging system identified 12 out of 135 patients (9 %) who had been identified as possible surgical candidates with N0/1 disease by radiological staging, with N2/3 disease, as confirmed by pathology. However, 11 (10 %) of the 114 surgical patients identified by ES were found to have N2 disease by surgical pathology. For these false-negative ES patients, it remains unclear if mediastinoscopy could have provided higher diagnostic accuracy for lymph nodes. In addition, the 5-year survival rate of this pN2 cohort (40.8 %) seemed better than the reported cN0pN2 prognosis (34 %) [15]. These results did not support the usefulness of EBUS-TBNA, but suggest ES underdiagnoses are acceptable, as they were not harmful for the patients.

**Fig. 2:**
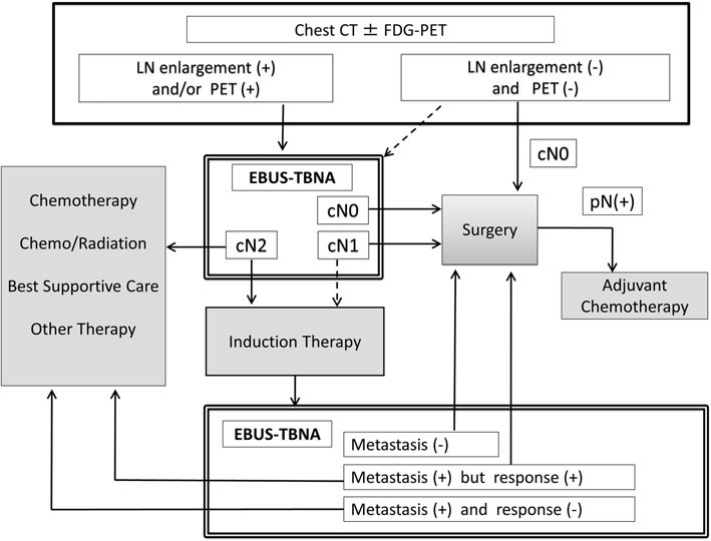
Strategy for identifying potentially resectable NSCLC. To more accurately determine N-stage, EBUS-TBNA was routinely performed in patients with radiologically node-positive disease or with a clinical status indicating potential for nodal metastasis. The modality is also applicable to the evaluation of treatment efficacy in potentially resectable N2 patients undergoing induction therapy

The staging and treatment algorithm for potentially resectable NSCLC treated at Chiba University Hospital is shown in Fig. 2. At our institution, EBUS-TBNA is an essential element for rescuing radiologically misdiagnosed operable cases according to N stage. It is especially notable that EBUS-TBNA can be repeatedly performed to evaluate the same target lesion; this function is very important for the timely evaluation of preoperative therapy (i.e., induction chemotherapy for N2 disease). Thus, restaging by EBUS-TBNA can identify patients who may benefit from trimodality treatment.

There are some limitations to this study. First, we did not perform FDG-PET in all patients; 58.3 % of enrolled patients (280/480) underwent preoperative FDG-PET. This study was a retrospective analysis, and the decision to add FDG-PET to the staging evaluation was decided according to the clinical information on each case. Fukui et al. [16] reported that patients with mediastinal lymph node metastasis evaluated by CT usually show a solid shadow in the lung without ground-glass opacities; therefore radiological findings may indicate that FDG-PET is needed for nodal staging. Furthermore, FDG-PET has been reported to have a high negative predictive value (97 %) and low positive predictive value (47 %) [17]; therefore, if lymph nodes are negative on FDG-PET, EBUS-TBNA may not be needed even if CT shows an enlarged mediastinal lymph node. It should be noted that adding PET for all lung cancer patients is not practical, especially with respect to medical economics at the present time. To determine optimized FDG-PET application criteria, we need further investigation with consideration to the cost−benefit.

The second limitation is that we did not perform EBUS-TBNA for all lymph node stations in each patient. We targeted nodes based on their size and progression of the primary lesion. A number of studies have used the criteria of nodal size as an indication for EBUS-TBNA, and a few patients with N2 disease were missed, including patients without mediastinal lymph node enlargement on CT who underwent EBUS-TBNA [18]. In order to reduce the chances of missing patients with N2 disease, we added 2 countermeasures for EBUS-TBNA: an extended indication and systematic nodal inspection. We increased the number of criteria used for selecting appropriate patients for EBUS-TBNA by including tumor size and a serum marker, but additional studies are needed to determine the criteria that enable EBUS-TBNA to be performed with maximum efficacy.

The third limitation is that we included EBUS-TBNA inaccessible nodes as #5, 6, 8, and 9 in the analyses. In this study, we wanted to compare the utility of pre-operative staging between clinical/radiological indicators and EBUS-TBNA. Hence, we included stations that are not accessible by EBUS-TBNA to clarify the efficacy of EBUS-TBNA in the daily clinical setting. We recognize that only 3 cases with pN2 disease proven by surgery from EBUS-TBNA inaccessible stations were included, but it does not affect the strong result of the final outcome.

## Conclusions

Radiological nodal staging of patients with NSCLC underestimates the stages in approximately 20 % of patients and overestimates the stages in 30 % of patients. The use of EBUS-TBNA leads to optimal treatment planning, with more accurate staging, and also identifies more surgical candidates.
